# Identification of Common Genes Refers to Colorectal Carcinogenesis with Paired Cancer and Noncancer Samples

**DOI:** 10.1155/2018/3452739

**Published:** 2018-01-30

**Authors:** Lihua Zhang, Yonglong Yang, Lin Cheng, Yu Cheng, Hong-Hao Zhou, Zhi-Rong Tan

**Affiliations:** ^1^Department of Clinical Pharmacology, Xiangya Hospital, Central South University, Changsha 410008, China; ^2^Institute of Clinical Pharmacology, Central South University, Hunan Key Laboratory of Pharmacogenetics, Changsha 410078, China; ^3^Haikou People's Hospital and Affiliated Haikou Hospital of Xiangya Medical School, Central South University, Haikou, Hainan 570311, China; ^4^State Key Laboratory of Ophthalmology, Zhongshan Ophthalmic Center, Sun Yat-sen University, 54 South Xianlie Road, Guangzhou, Guangdong, China

## Abstract

Colorectal cancer is a malignant tumor which harmed human beings' health. The aim of this study was to explore common biomarkers associated with colorectal carcinogenesis in paired cancer and noncancer samples. At first, fifty-nine pairs of colorectal cancer and noncancer samples from three gene expression datasets were collected and analyzed. Then, 181 upregulation and 282 downregulation common differential expression genes (DEGs) were found. Next, functional annotation was performed in the DAVID database with the DEGs. Finally, real-time polymerase chain reaction (PCR) assay was conducted to verify the analyses in sixteen colorectal cancer and individual-matched adjacent mucosa samples. Real-time PCR showed that *MCM2*, *RNASEH2A*, and *TOP2A* were upregulated in colorectal cancer compared with adjacent mucosa samples (*MCM2*, *P* < 0.001; *RNASEH2A*, *P* < 0.001; *TOP2A*, *P* = 0.001). These suggested that 463 DEGs might contribute to colorectal carcinogenesis.

## 1. Introduction

Colorectal cancer is one of the most commonly diagnosed cancers worldwide, with approximately 1.4 million cases and 693,900 deaths in 2012 [1]. Some risk factors may lead to colorectal cancer such as family history [2, 3], inflammatory bowel disease [4], and smoking [5, 6]. In the molecular level, colorectal cancer is a heterogeneous disease. Mutations, epigenetic changes, and expression differences of multiple genes are well-known colorectal cancer contributors. However, the underlying molecular mechanism of colorectal carcinogenesis has not been fully understood yet.

More than twenty years ago, complementary DNA microarray has been used to analyze the gene expression patterns in human cancer [7]. With the development of technology, more and more expression profile platforms and researches on colorectal cancer have been released in recent years. Currently, a large number of expression profile datasets were uploaded and shared publically on several public databases, and Gene Expression Omnibus (GEO, https://www.ncbi.nlm.nih.gov/geo) and ArrayExpress database (http://www.ebi.ac.uk/arrayexpress) were included. The public shared data of the databases could benefit the researchers greatly in finding interesting research targets or verify their ideas easily.

In this study, to further understand the mechanism of colorectal carcinogenesis from the gene expression level, we searched the GEO database for paired colorectal cancer and noncancer samples. Fifty-nine paired samples from GSE21510, GSE23878, and GSE32323 were selected in total. The DEGs were screened from three datasets, and 463 common DEGs (181 upregulation and 282 downregulation genes) were detected and sent to the DAVID database (https://david.ncifcrf.gov) for functional annotation. Three upregulated genes were chosen to verify the analysis results in sixteen pairs of colorectal cancer and adjacent mucosa tissues. Real-time PCR results showed that *MCM2*, *RNASEH2A*, and *TOP2A* were associated with colorectal carcinogenesis.

## 2. Materials and Methods

### 2.1. Microarray Data

We searched the NCBI-GEO database with the following keywords: Human Genome U133 Plus 2.0, colorectal cancer, and normal (Human Genome U133 Plus 2.0 Array is a platform of Affymetrix). Fifty-nine paired colorectal cancer and noncancerous samples were selected from three gene expression datasets (twenty-three pairs in GSE21510 from Japan, seventeen pairs in GSE23878 from Saudi Arabia, and nineteen pairs in GSE32323 from Japan). The cancer and noncancerous samples were divided into cancer and noncancer groups, and the noncancer group was used as control.

### 2.2. Identification of DEGs

The selected samples of GSE21510, GSE23878, and GSE32323 were separately analyzed by R software (https://www.r-project.org). At first, raw data files of the three datasets were downloaded from the GEO database. Then, the Robust Multichip Average algorithm was applied to perform background correction and quantile normalization with the R package Affy [8, 9]. Next, the probability of genes being differentially expressed between cancer and noncancer groups were calculated by the Linear Models for Microarray Data (LIMMA) package. Finally, the DEGs were selected under corrected *P* < 0.05 and |fold change| ≥ 2.0 criteria. The volcano plots of differentially expressed genes were also performed by R software [10]. The DEGs from each dataset were intersected to identify common DEGs.

### 2.3. Gene Ontology and Pathway Enrichment Analysis of DEGs

Gene Ontology (GO, http://www.geneontology.org) was a framework for the model of biology that describes the attributes of gene products. It was classified into three aspects: molecular function, biological process, and cellular component [11]. Pathway analysis was a popular method to analyze microarray data in a more detailed, specific way [12, 13]. DAVID database was a free online bioinformatics resource that aimed to provide functional interpretation of large lists of genes derived from genomic studies [14]. The common DEGs were input in the DAVID database to perform the GO and pathway analysis [15]. *P* < 0.05 was considered statistically significant.

### 2.4. Patients and Tissue Specimens

Tumor tissue samples and individual-matched adjacent mucosa samples were obtained from sixteen patients with colorectal cancer who underwent resection at Xiangya Hospital between 2014 and 2016, and the adjacent mucosa samples were acquired 2–5 cm away from the tumor. The dissected tissue samples were collected in the operating room and stored immediately in liquid nitrogen. This study was approved by the Institutional Review Board of Department of Clinical Pharmacology, Xiangya Hospital, Central South University (registration number: CTXY-150001-2) and by Chinese Clinical Trial Registry (registration number: ChiCTR-DCD-15006289).

### 2.5. RNA Isolation, Reverse Transcription, and Real-Time PCR

Tissue specimens were grounded and added with TRIzol reagent (Takara). Then the total RNA was isolated, and 1 *μ*g of RNA was reverse-transcripted with PrimeScript 1st Strand complementary DNA Synthesis kit (Takara). Real-time PCR assay was performed on ABI 7500 platform. SYBR Premix Dimer Eraser kit (Takara) was used in 20 *μ*l reaction volume, and the cycling conditions were as follows: an initial 30 s denaturation at 95°C and 45 cycles (5 s at 95°C, 30 s at 55°C, and 34 s at 72°C). *PPIA* and *B2M* genes were set as internal controls. *MCM2*, *RNASEH2A*, and *TOP2A* expression level was detected in sixteen pairs of colorectal cancer and adjacent mucosa samples. The primer sequences were shown in Supplementary Table 1.

### 2.6. Statistical Analysis

Statistical analyses were performed with SPSS version 18.0 (IBM Corporation) and GraphPad Prism 6.0 software (GraphPad Software Inc.). DEGs were determined using t-statistics from the LIMMA Bioconductor package. The real-time PCR data was analyzed with 2^−ΔCt^ and the significance of the difference between the cancer and noncancer groups was evaluated by two-tailed Student's *t*-test. *P* values less than 0.05 were considered statistically significant.

## 3. Results

### 3.1. Identification of DEGs

**Figure 1:**
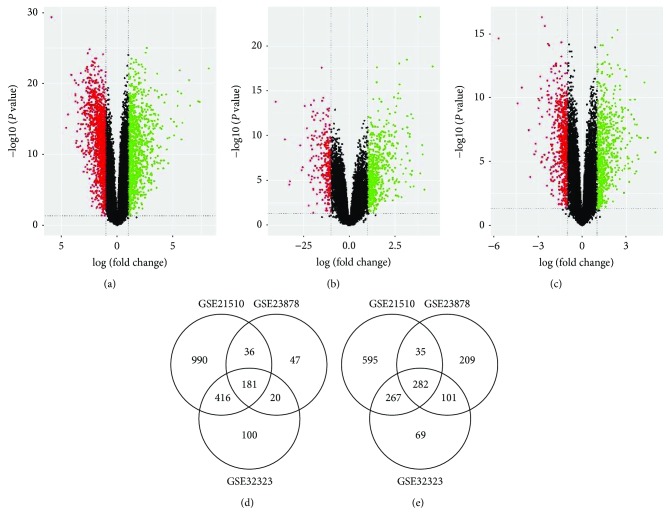
Identification of DEPs and DEGs between tumor and nontumor samples. (a–c) Volcano plot of the differential mRNA expression analysis. *x*-axis: log2 fold change; *y*-axis: −log10 (FDR *P* value) for each probe; vertical dotted lines: fold change ≥ 2 or ≤2; horizontal dotted line: the significance cutoff (FDR *P* value = 0.05). (a) There were 2802 genes identified to be differentially expressed in GSE21510, including 1623 upregulated and 1179 downregulated genes. (b) There were 911 genes identified to be differentially expressed in GSE23878, including 284 upregulated and 627 downregulated genes. (c) There were 1436 genes identified to be differentially expressed in GSE32323, including 717 upregulated and 719 downregulated genes. (d–e) Overlap analysis of upregulated genes and downregulated genes between different datasets. (d) A total of 181 genes were significantly upregulated in the three colorectal cancer datasets. (e) A total of 282 genes were significantly downregulated in the three colorectal cancer datasets.

Analysis results showed 1623 upregulated and 1179 downregulated DEGs in GSE21510 (Figure 1(a)), 284 upregulated and 627 downregulated DEGs in GSE23878 (Figure 1(b)), and 717 upregulated and 719 downregulated DEGs (Figure 1(c)) in GSE32323. Then, the DEGs of the three datasets were merged. 463 common DEGs were found in total (Supplementary Table 1), consisting of 181 upregulated and 282 downregulated genes (Figures 1(d)–1(e)).

### 3.2. GO Term Enrichment Analysis

The 181 upregulated genes were uploaded to the DAVID database for GO analysis. The results showed that upregulated DEGs were significantly enriched in biological process, including 22 DEGs in cell division (*P* = 1.48*E* − 11) and 19 in mitotic nuclear division (*P* = 2.06*E* − 11) GO terms. In the cellular component, nucleoplasm (*P* = 4.95*E* − 07), spindle microtubule (*P* = 2.49*E* − 06), and spindle pole (*P* = 6.09*E* − 06) were the top three GO terms, and there were 51 DEGs enriched in the nucleoplasm. In molecular function, the top three GO terms were frizzled binding (*P* = 3.70*E* − 04), protein binding (*P* = 4.65*E* − 04), and microtubule binding (*P* = 8.73*E* − 04) (Table 1).

The 282 downregulated DEGs were also imported into the DAVID database. GO analysis results showed that the top three terms in biological process were bicarbonate transport (*P* = 2.88*E* − 06), negative regulation of growth (*P* = 5.88*E* − 06), and cellular response to zinc ion (*P* = 5.88*E* − 06). Extracellular exosome (*P* = 4.45*E* − 08), extracellular space (*P* = 2.88*E* − 06), and bicarbonate transport (*P* = 8.19*E* − 05) in cellular component were the top three GO terms. In molecular function, carbonate dehydratase activity (*P* = 3.51*E* − 05), chloride channel activity (*P* = 1.11*E* − 04), and oxidoreductase activity (*P* = 0.0022) were the top three GO terms (Table 1).

### 3.3. KEGG Pathway Analysis

After all the DEGs were input into the DAVID database, KEGG pathway analysis results were also acquired. In Table 2, the most significantly enriched pathway of the upregulated and downregulated pathways was set out.

The upregulated DEGs were significantly enriched in the cell cycle (*P* = 2.90*E* − 07), p53 signaling pathway (*P* = 0.028), and DNA replication pathways (*P* = 0.049) (Table 2). The downregulated DEGs were enriched in mineral absorption (*P* = 8.10*E* − 07), nitrogen metabolism (*P* = 1.57*E* − 04), bile secretion (*P* = 0.001), retinol metabolism (*P* = 0.005), proximal tubule bicarbonate reclamation (*P* = 0.007), pancreatic secretion (*P* = 0.020), pentose and glucuronate interconversions (*P* = 0.023), renin secretion (*P* = 0.023) pathways, and so forth (Table 2).

### 3.4. Real-Time PCR Validation of DEGs

**Figure 2:**
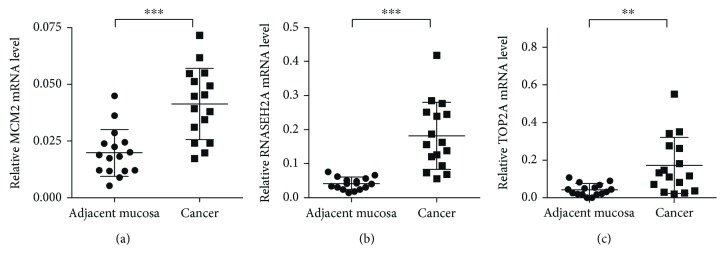
Validation of the differentially expressed genes. (a) Validation of mRNA expression of *MCM2* expression in sixteen colorectal cancer and individual-matched adjacent mucosa samples. *MCM2* expression level in colorectal cancer samples was significantly higher than that in adjacent mucosa samples (*P* < 0.001). (b) Validation of mRNA expression of *RNASEH2A* in sixteen colorectal cancer and individual-matched adjacent mucosa samples. *RNASEH2A* expression level in colorectal cancer samples was significantly higher than that in adjacent mucosa samples (*P* < 0.001). (c) Validation of mRNA expression of *TOP2A* in sixteen colorectal cancer and individual-matched adjacent mucosa samples. *TOP2A* expression level in colorectal cancer samples was significantly higher than that in adjacent mucosa samples (*P* = 0.001). ^∗∗^ *P* < 0.01 and ^∗∗∗^ *P* < 0.001.

To test the DEGs of the analysis, *MCM2*, *RNASEH2A*, and *TOP2A* were chosen to conduct real-time PCR in sixteen pairs of colorectal cancer and adjacent mucosa samples. *MCM2*, *RNASEH2A*, and *TOP2A* expression level in cancer samples was significantly higher than noncancer samples (*MCM2*, *P* < 0.001, Figure 2(a); *RNASEH2A*, *P* < 0.001, Figure 2(b); *TOP2A*, *P* = 0.001, Figure 2(c)).

## 4. Discussion

Colorectal cancer was a complex disease. To get more information for colorectal cancer occurrence, the gene expression data of fifty-nine paired colorectal cancer and noncancer tissues was extracted from the GSE21510, GSE23878, and GSE32323 datasets. The fifty-nine paired samples were from two countries: Japan (40 samples from SE21510 and GSE32323) and Saudi Arabia (19 samples from SE32323). The regional divergence of the samples might contribute to finding the common DEGs from two the ethnic groups.

After analysis, 181 upregulated DEGs and 282 downregulated common DEGs were screened. Pathway analyses showed that the upregulated DEGs were mainly involved in cell cycle pathway (*n* = 11), p53 signaling pathway (*n* = 4), and DNA replication pathway (*n* = 3).

Cell cycle plays a crucial role in tumor evolution and progression, so it acts as an important target for antitumor drugs, such as paclitaxel, vincristine, and Adriamycin. In this study, 3 genes (CDK1, CDK4, and CCNB1) refer to cell cycle pathway were also included in the p53 signaling pathway. CDK1 and CDK4 were key protein kinase for cell cycle control [16, 17]. *CCNB1* was also a regulatory protein involved in mitosis [18]. In the p53 signaling pathway, the RRM2 gene was an oncogene that overexpressed in colorectal cancer, with its elevated expression correlated with invasion depth, poorly differentiated type, and tumor node metastasis stage [19]. Transcription factor *E2F1* could promote *RRM2* expression in colorectal cancer cell lines [20].

DNA replication is an important pathway in carcinogenesis, which ranked the third in upregulated DEGs. Three upregulated DEGs refer to DNA replication were *MCM2*, *RFC3*, and *RNASEH2A*. *MCM2* was a member of the *MCM* family (*MCM2-7*), and all 6 members of this family could form a hexameric protein complex with each other. This complex worked as a DNA helicase to untie the DNA double helix at the initiation stage of DNA synthesis [21]. *MCM2* expression was reported to be associated with colorectal cancer stage and prognosis [22] and used to detect colorectal cancer in stool [23].

Eukaryotic RNase H2 was a heterotrimeric enzyme formed by *RNASEH2A*, *RNASEH2B*, and *RNASEH2C*. *RNASEH2A* was a catalytic subunit that could hydrolyze RNA/DNA hybrid substrate with the structural support from *RNASEH2B* and *RNASEH2C* subunits [24]. It had been reported that *RNASEH2A* showed higher expression level in colorectal cancer [25], and this was validated in our research.

*TOP2A* was a gene that involves copy number variation and chromosomal instability in many cancers [26–29]. In colorectal cancer, protein expression level of *TOP2A* was related to aggressive tumor phenotype and advanced tumor stage [30]. In our research, we found that *TOP2A* mRNA expression level was upregulated in colorectal cancer.

The downregulated DEGs are mainly involved in mineral absorption, nitrogen metabolism, bile secretion, retinol metabolism, proximal tubule bicarbonate reclamation, pancreatic secretion and so on, which may signal that the tumor cells lose some function for metabolism of the normal colorectal epithelial cell.

In real-time PCR assay, the commonly used internal genes were *GAPDH*, *β-actin*, *tubulin*, and so forth. However, in colorectal cancer tissues, *GAPDH* was not a good internal control gene since it showed higher transcription level than in normal mucosa, nor was *β-actin* [31, 32]. The combined application of *B2M* and *PPIA* were better internal control than others in colorectal cancer [33]. Therefore, *B2M* and *PPIA* were used as internal control in our study.

In summary, these gene expression profile analysis results suggested 463 candidate biomarkers for early screening and diagnosis of colorectal cancer. Our study confirmed that *MCM2*, *RNASEH2A*, and *TOP2A* were upregulated in colorectal cancer. The protein expression level and functional studies of these markers were warranted to reveal the molecular mechanism of colorectal cancer development.

## Figures and Tables

**Table 1:** Go analysis of DEGs between paired tumor and nontumor sample.

Expression	Category	Term/gene function	Count	%	*P* value	
Upregulated	GOTERM_BP_DIRECT	GO:0051301~cell division	22	8.25	1.48*E* − 11
	GOTERM_BP_DIRECT	GO:0007067~mitotic nuclear division	19	7.13	2.06*E* − 11
	GOTERM_BP_DIRECT	GO:0000281~mitotic cytokinesis	7	2.63	2.51*E* −07
	GOTERM_BP_DIRECT	GO:0030574~collagen catabolic process	8	3.00	2.26*E* − 06
	GOTERM_BP_DIRECT	GO:0006260GO:0000082~G1/S transition of mitotic cell cycle	9	3.38	5.34*E* − 06
	GOTERM_CC_DIRECT	GO:0005654~nucleoplasm	51	19.13	4.95*E* − 07
	GOTERM_CC_DIRECT	GO:0005876~spindle microtubule	7	2.63	2.49*E* − 06
	GOTERM_CC_DIRECT	GO:0000922~spindle pole	9	3.38	6.09*E* − 06
	GOTERM_CC_DIRECT	GO:0005819~spindle	8	3.00	1.08*E* − 04
	GOTERM_CC_DIRECT	GO:0005578~proteinaceous extracellular matrix	11	4.13	1.53*E* − 04
	GOTERM_MF_DIRECT	GO:0005109~frizzled binding	5	1.88	3.70*E* − 04
	GOTERM_MF_DIRECT	GO:0005515~protein binding	106	39.75	4.65*E* − 04
	GOTERM_MF_DIRECT	GO:0008017~microtubule binding	9	3.38	8.73*E* − 04
	GOTERM_MF_DIRECT	GO:0019901~protein kinase binding	12	4.50	9.80*E* − 04
	GOTERM_MF_DIRECT	GO:0008009~chemokine activity	5	1.88	0.0012

Downregulated	GOTERM_BP_DIRECT	GO:0015701~bicarbonate transport	8	2.13	2.88*E* − 06
	GOTERM_BP_DIRECT	GO:0045926~negative regulation of growth	6	1.60	5.88*E* − 06
	GOTERM_BP_DIRECT	GO:0071294~cellular response to zinc ion	6	1.60	5.88*E* − 06
	GOTERM_BP_DIRECT	GO:0006730~one-carbon metabolic process	6	1.60	6.33*E* − 05
	GOTERM_BP_DIRECT	GO:0007586~digestion	7	1.87	2.88*E* − 04
	GOTERM_CC_DIRECT	GO:0070062~extracellular exosome	75	20.00	4.45*E* − 08
	GOTERM_CC_DIRECT	GO:0005615~extracellular space	42	11.20	2.88*E* − 06
	GOTERM_CC_DIRECT	GO:0031526~brush border membrane	7	1.87	8.19*E* − 05
	GOTERM_CC_DIRECT	GO:0048471~perinuclear region of cytoplasm	19	5.07	0.0035
	GOTERM_CC_DIRECT	GO:0016021~integral component of membrane	93	24.81	0.0067
	GOTERM_MF_DIRECT	GO:0004089~carbonate dehydratase activity	5	1.33	3.51*E* − 05
	GOTERM_MF_DIRECT	GO:0005254~chloride channel activity	7	1.87	1.11*E* − 04
	GOTERM_MF_DIRECT	GO:0016491~oxidoreductase activity	10	2.67	0.0022
	GOTERM_MF_DIRECT	GO:0008201~heparin binding	8	2.13	0.0080
	GOTERM_MF_DIRECT	GO:0005179~hormone activity	6	1.60	0.0108

**Table 2:** Pathway analysis of DEGs between paired tumor and nontumor samples.

Expression	Pathway	Count	Term/gene function	%	*P* value
Upregulated	hsa04110: Cell cycle	11	CCNB1, CDK1, CDC6, MAD2L1, TTK, BUB1B, ORC6, MCM2, PTTG1, CDK4, CDC25B	4.13	2.90*E* − 07
	hsa04115: p53 signaling pathway	4	CCNB1, CDK1, RRM2, CDK4	1.50	0.028
	hsa03030: DNA replication	3	RFC3, MCM2, RNASEH2A	1.13	0.049

Downregulated	hsa04978: mineral absorption	9	SLC26A3, TRPM6, MT1M, MT2A, CYBRD1, MT1E, MT1H, MT1X, MT1F	2.40	8.10*E* − 07
	hsa00910: nitrogen metabolism	5	CA12, CA7, CA4, CA2, CA1	1.33	1.57*E* − 04
	hsa04976: bile secretion	7	AQP8, PRKACB, SLC51B, CA2, SLC51A, SLC4A4, ABCG2	1.87	0.001
	hsa00830: retinol metabolism	6	ALDH1A1, ADH1C, DHRS9, ADH1B, UGT2A3, RETSAT	1.60	0.005
	hsa04964: proximal tubule Bicarbonate reclamation	4	CA4, CA2, SLC4A4, PCK1	1.07	0.007
	hsa04972: pancreatic secretion	6	SLC26A3, CLCA1, CLCA4, PLA2G10, CA2, SLC4A4	1.60	0.020
	hsa00040: pentose and glucuronate interconversions	4	AKR1B10, UGDH, UGT2A3, UGP2	1.07	0.023
	hsa04924: renin secretion	5	CLCA1, CLCA4, GNAI1, PDE3A, PRKACB	1.33	0.023
	hsa04960: aldosterone-regulated sodium reabsorption	4	SGK1, NR3C2, HSD11B2, SCNN1B	1.07	0.028
	hsa04530: tight junction	7	CLDN8, EPB41L3, GNAI1, MYH11, JAM2, CLDN23, MYL9	1.87	0.028
	hsa05204: chemical carcinogenesis	5	NAT2, ADH1C, ADH1B, SULT1A2, UGT2A3	1.33	0.046
	hsa04670: leukocyte transendothelial migration	6	CLDN8, GNAI1, JAM2, CXCL12, CLDN23, MYL9	1.60	0.049
	hsa05030: cocaine addiction	4	GNAI1, MAOA, PRKACB, FOSB	1.07	0.050

## Supplementary Materials

Supplementary Materials Part. 1. Primer sequences for real-time PCR. Part. 2. 181 upregulated and 282 downregulated common differential expressed genes in colorectal cancer samples.
