# Supplementary material for: Identification of Common Genes Refers to Colorectal Carcinogenesis with Paired Cancer and Noncancer Samples
Source: Dis Markers. 2018 Jan 30;2018:3452739. doi: 10.1155/2018/3452739 (PMC5830953; doi:10.1155/2018/3452739)
Supplement: Supplementary Materials — Part. 1. Primer sequences for real-time PCR. Part. 2. 181 upregulated and 282 downregulated common differential expressed genes in colorectal cancer samples. [file 3452739.f1.docx]

Part. 1. Primer sequences for real-time PCR.

| Gene name | Primer sequence |
| --- | --- |
| PPIA | F: 5^，^-GTGGTATAAAAGGGGCGGGAG- 3^，^ |
|  | R: 5^，^-GTGGGGTTGACCATGGCTAATAGTA- 3^，^ |
| B2M | F: 5^，^-ATGCCTGCCGTGTGAACCATGTGA- 3^，^ |
|  | R: 5^，^-TCCAAATGCGGCATCTTCAAACCTC- 3^，^ |
| MCM2 | F: 5^，^-ATGGCGGAATCATCGGAATCC- 3^，^ |
|  | R: 5^，^-GGTGAGGGCATCAGTACGC- 3^，^ |
| RNASEH2A | F: 5^，^-ACAGCCACTGGGCTTATACAG- 3^，^ |
|  | R: 5^，^-TCCCTACGGTGTCCACGAATA- 3^，^ |
| TOP2A | F: 5^，^-CTAGTTAATGCTGCGGACAACA- 3^，^  R: 5^，^-CATTTCGACCACCTGTCACTT- 3^，^ |

Part. 2 181 up-regulated and 282 down-regulated common differential expressed genes in colorectal cancer samples.

| Up-regulated genes | Down-regulated genes |
| --- | --- |
| FOXQ1 | DDX60 |
| MMP1 | MRGPRF |
| CTHRC1 | LMOD1 |
| EPHX4 | PLN |
| NUF2 | ABI3BP |
| TGFBI | RCAN2 |
| RAD51AP1 | ANGPTL1 |
| ANLN | PLP1 |
| MMP12 | TOX |
| VSNL1 | RSPO3 |
| MMP3 | FXYD6 |
| CXCL1 | SDPR |
| KRT23 | MYL9 |
| TTK | ABCA8 |
| CRNDE | FHL1 |
| SLC39A10 | MYH11 |
| TDGF1P3///TDGF1 | ST6GALNAC1 |
| DACH1 | MFAP4 |
| MAD2L1 | SPARCL1 |
| CLDN1 | SUPT20H///DES |
| HMMR | SCGN |
| NUDCD1 | FGL2 |
| NFE2L3 | SST |
| DPEP1 | TAGLN |
| DLGAP5 | SYNPO2 |
| UBE2T | IGHM |
| SPC25 | SOCS6 |
| CEP55 | PLSCR4 |
| CXCL3 | CCL19 |
| C2CD4A | PDK4 |
| EGFL6 | TCL1A |
| CDK1 | LOC100287411 |
| CYP4X1 | CASQ2 |
| KIF15 | PELI2 |
| LOC100505730 | ITM2A |
| WNT5A | STMN2 |
| CDKN3 | PYY |
| FAM72A///FAM72D///FAM72B///FAM72C | GREM2 |
| NCAPG | IGFBP6 |
| TRIP13 | PRKAR2B |
| MMP7 | SMPDL3A |
| ZNRF3 | PDE3A |
| CKS2 | RAB27A |
| COL11A1 | UGP2 |
| CCNB1 | ABCC13 |
| RNF183 | SCGB2A1 |
| RPP40 | RERGL |
| RAD54B | KCTD9 |
| HS6ST2 | NDN |
| LY6G6D | PRIMA1 |
| KIF20A | SLC22A5 |
| CEMIP | FAM107A |
| PRMT3 | GNAI1 |
| CLDN2 | NKX2-3 |
| KIF18A | PLCL2 |
| ATAD2 | PRSS12 |
| AZGP1 | CNN1 |
| ZFAS1 | DUSP5 |
| INHBA | FAM107B |
| SLC7A5 | EIF4E3 |
| KIF4A | SCG2 |
| CXCL8 | CSRP1 |
| CMSS1 | GBP3 |
| SGOL2 | FXYD3 |
| TPX2 | MEIS3P1 |
| BUB1B | NR5A2 |
| CSE1L | MIER3 |
| PPIL1 | ETFDH |
| CDH3 | KRT24 |
| CXCL11 | C15orf48 |
| LRP8 | PDCD4 |
| DUSP14 | LPAR1 |
| GTF3A | FDCSP |
| OSBPL3 | KIAA1211 |
| ZNF239 | HHLA2 |
| NME1 | GALNT12 |
| RFC3 | FBXO32 |
| KIF11 | CILP |
| XPOT | DPT |
| DTL | ADRA2A |
| CKMT2 | BCHE |
| SLCO1B3 | PRKACB |
| RRM2 | SCARA5 |
| TOP2A | CCL15-CCL14///CCL15 |
| WDR12 | ZBTB16 |
| PRC1 | ARRDC4 |
| BORA | JAM2 |
| PSAT1 | SLC41A2 |
| AGT | HIGD1A |
| RNF43 | UGDH |
| UBE2C | MFSD4 |
| PUS7 | SEMA6D |
| FIGNL1 | SYNC |
| GINS1 | ALDH1A1 |
| WDR43 | SULT1B1 |
| LRP11 | NXPE1 |
| TCFL5 | TRANK1 |
| NUSAP1 | PCOLCE2 |
| SLC35D3 | MBNL1-AS1 |
| NANP | THRB |
| MAP7D2 | FOSB |
| NCAPG2 | GHR |
| TEX30 | CYBRD1 |
| LOC729680 | RBM24 |
| JADE3 | LOC344887 |
| DKC1 | CCL15-CCL14///CCL14 |
| C5orf34 | CXCL12 |
| DDIAS | MOGAT2 |
| RASSF10 | AGR3 |
| PDCD2L | SQRDL |
| PNPT1 | STYK1 |
| PTTG1 | MFAP5 |
| AJUBA | MIR22HG |
| SOX9 | GNE |
| HILPDA | MBOAT1 |
| RACGAP1 | SCIN |
| ASCL2 | C7 |
| SKA3 | C4orf19 |
| RUVBL1 | NAT2 |
| APCDD1 | SEMA6A |
| MACC1 | LINC01279 |
| SNHG17 | C2orf40 |
| TCN1 | PIGR |
| NMU | SPON1 |
| PPBP | MAMDC2 |
| AHCY | FOS |
| ZNF713///MRPS17 | CAPN9 |
| RNASEH2A | PAG1 |
| CDC25B | ENDOD1 |
| GPSM2 | EMP1 |
| AURKA | MAOA |
| ERP27 | UGT2A3 |
| CDC6 | CHGA |
| GTF2IRD1 | RNF125 |
| CIRH1A | NAAA |
| MCM2 | IGHA2///IGHA1 |
| TMEM97 | SPPL2A |
| LOC100506918 | CLU |
| SCD | MXD1 |
| UBD///GABBR1 | BTNL3 |
| ORC6 | MEP1B |
| BBOX1-AS1 | KRT20 |
| LRRC6 | GPX3 |
| CDK4 | FABP1 |
| CENPA | PAPSS2 |
| PSMG1 | ADH1B |
| ZIC2 | SIDT1 |
| SP5 | CNTN3 |
| MRGBP | MEP1A |
| CEL | VIPR1 |
| HSPH1 | RUNDC3B |
| PMEPA1 | CES3 |
| TMEM158 | PARM1 |
| WNT2 | VIP |
| CD3EAP | ISX |
| S100A2 | PIGZ |
| ESM1 | TTLL6 |
| TTI1 | ARL14 |
| GGCT | FKBP1B |
| CDCA3 | EDN3 |
| KLK6 | ENTPD5 |
| FXYD5 | ACKR1 |
| KRT80 | SULT1A2 |
| SHMT2 | GCNT2 |
| MMP10 | C10orf99 |
| SLC5A6 | FRMD3 |
| COL10A1 | OGN |
| ETV4 | CCDC68 |
| C19orf48 | APPL2 |
| GPR143 | PPAP2A |
| TEAD4 | IL6R |
| CDCA5 | TMEM171 |
| DGAT2 | IL1R2 |
| NELFCD | CLDN23 |
| TRIB3 | CFD |
| GALNT6 | CCL28 |
| NPTX2 | XDH |
| BYSL | AHCYL2 |
| TOMM34 | CPM |
| NOP2 | MYO1A |
| LAPTM4B | TRPM6 |
|  | SFRP2 |
|  | GGT6 |
|  | LGALS2 |
|  | MT1HL1 |
|  | SRPX |
|  | TMCC3 |
|  | GPA33 |
|  | SLC26A3 |
|  | MMP28 |
|  | MT2A |
|  | CDKN2B |
|  | MT1X |
|  | ITLN1 |
|  | LOC100505584///MT1E |
|  | TMEM100 |
|  | SLC44A4 |
|  | CXCL13 |
|  | PCK1 |
|  | RETSAT |
|  | ST6GALNAC6 |
|  | MT1F |
|  | CLEC3B |
|  | GCG |
|  | CA12 |
|  | CKB |
|  | SLC4A4 |
|  | UGT1A1///UGT1A4///UGT1A9///UGT1A6///UGT1A8///UGT1A10 |
|  | BEST2 |
|  | SGK1 |
|  | SLC26A2 |
|  | PLA2G10 |
|  | NXPE4 |
|  | HMGCS2 |
|  | NPY1R |
|  | SLCO2A1 |
|  | GCNT3 |
|  | MT1H |
|  | HPGD |
|  | HSD11B2 |
|  | CHRDL1 |
|  | TUBAL3 |
|  | BMP2 |
|  | UGT1A3///UGT1A1///UGT1A4///UGT1A9///UGT1A5///UGT1A6///UGT1A7///UGT1A8///UGT1A10 |
|  | GDPD3 |
|  | NR3C2 |
|  | TMEM220 |
|  | MT1E |
|  | ANO5 |
|  | HEPACAM2 |
|  | SLC16A9 |
|  | SPINK5 |
|  | AGPAT9 |
|  | KLF4 |
|  | SDCBP2 |
|  | ITM2C |
|  | TSPAN1 |
|  | TSPAN7 |
|  | CLCA1 |
|  | GBA3 |
|  | CA1 |
|  | TP53INP2 |
|  | ADTRP |
|  | CWH43 |
|  | AOC1 |
|  | CA7 |
|  | LRRC19 |
|  | PLAC8 |
|  | VSIG2 |
|  | EPB41L3 |
|  | CDHR5 |
|  | BTNL8 |
|  | SLC51A |
|  | DHRS11 |
|  | CHP2 |
|  | ADH1C |
|  | SLC51B |
|  | SCNN1B |
|  | BEST4 |
|  | FCGBP |
|  | MUC2 |
|  | MALL |
|  | C2orf88 |
|  | HSD17B2 |
|  | AKR1B10 |
|  | SI |
|  | PKIB |
|  | SLC30A10 |
|  | CEACAM7 |
|  | ABCG2 |
|  | DHRS9 |
|  | GUCA2B |
|  | CD177 |
|  | GUCA2A |
|  | MT1M |
|  | CLDN8 |
|  | CA2 |
|  | CA4 |
|  | ZG16 |
|  | MS4A12 |
|  | AQP8 |
|  | CLCA4 |
